# The Methanol Poisoning Outbreaks in Libya 2013 and Kenya 2014

**DOI:** 10.1371/journal.pone.0152676

**Published:** 2016-03-31

**Authors:** Morten Rostrup, Jeffrey K. Edwards, Mohamed Abukalish, Masoud Ezzabi, David Some, Helga Ritter, Tom Menge, Ahmed Abdelrahman, Rebecca Rootwelt, Bart Janssens, Kyrre Lind, Raido Paasma, Knut Erik Hovda

**Affiliations:** 1 Department of Acute Medicine, Oslo University Hospital, Oslo, Norway; 2 Médecins Sans Frontières International, Geneva, Switzerland; 3 Institute of Basic Medical Sciences, University of Oslo, Oslo, Norway; 4 Médecins Sans Frontières, Nairobi, Kenya; 5 Department of International Health, School of Public Health, Johns Hopkins University, Baltimore, Maryland, United States of America; 6 Libyan Emergency Medicine Association, Tripoli Medical Center, Tripoli, Libya; 7 Medical Department, Tripoli Central Hospital, Tripoli, Libya; 8 Department of Pharmacy, Kenyatta National Hospital, Nairobi, Kenya; 9 Médecins Sans Frontières, Libya Mission, Tripoli, Libya; 10 Médecins Sans Frontières Operational Centre, Brussels, Belgium; 11 Médecins Sans Frontières, Oslo, Norway; 12 Department of Anesthesiology and ICU, Pärnu County Hospital, Pärnu, Estonia; 13 The Norwegian CBRNe Centre of Medicine, Department of Acute Medicine, Oslo University Hospital, Oslo, Norway; Nottingham University, UNITED KINGDOM

## Abstract

**Background:**

Outbreaks of methanol poisoning occur frequently on a global basis, affecting poor and vulnerable populations. Knowledge regarding methanol is limited, likely many cases and even outbreaks go unnoticed, with patients dying unnecessarily. We describe findings from the first three large outbreaks of methanol poisoning where Médecins Sans Frontières (MSF) responded, and evaluate the benefits of a possible future collaboration between local health authorities, a Non-Governmental Organisation and international expertise.

**Methods:**

Retrospective study of three major methanol outbreaks in Libya (2013) and Kenya (May and July 2014). Data were collected from MSF field personnel, local health personnel, hospital files, and media reports.

**Findings:**

In Tripoli, Libya, over 1,000 patients were poisoned with a reported case fatality rate of 10% (101/1,066). In Kenya, two outbreaks resulted in approximately 341 and 126 patients, with case fatality rates of 29% (100/341) and 21% (26/126), respectively. MSF launched an emergency team with international experts, medications and equipment, however, the outbreaks were resolving by the time of arrival.

**Interpretation:**

Recognition of an outbreak of methanol poisoning and diagnosis seem to be the most challenging tasks, with significant delay from time of first presentations to public health warnings being issued. In spite of the rapid response from an emergency team, the outbreaks were nearly concluded by the time of arrival. A major impact on the outcome was not seen, but large educational trainings were conducted to increase awareness and knowledge about methanol poisoning. Based on this training, MSF was able to send a local emergency team during the second outbreak, supporting that such an approach could improve outcomes. Basic training, simplified treatment protocols, point-of-care diagnostic tools, and early support when needed, are likely the most important components to impact the consequences of methanol poisoning outbreaks in these challenging contexts.

## Introduction

Methanol is a common organic solvent mainly used for industrial purposes. It is sometimes mixed with ethanol in alcoholic beverages either by mistake or more commonly, as an inexpensive substitute for ethanol to increase profit. Despite effective treatment, morbidity and mortality from methanol remains high, disproportionately affecting people in the developing countries,[1–7] but also regularly seen in the developed part of the world.[8–10]

Symptoms usually appear within 12–24 hours after ingestion, but can be significantly delayed if ethanol is ingested simultaneously. Methanol itself is not toxic, but is metabolized by alcohol dehydrogenase to formic acid, which is responsible for the severe toxicity. Clinical features are nonspecific, with gastrointestinal symptoms, dyspnoea, chest pain and hyperventilation dominating along with visual disturbances.[11, 12] The lack of characteristic symptoms highlights the importance of analytical tools for diagnosis, which are absent in most places. Furthermore, limited knowledge of basic toxicological principles in areas where these poisonings are endemic, leaves many victims untreated and without a correct diagnosis. Incidents where dozens or even hundreds of patients are affected, are often solely reported in the media.[13, 14]

Sodium bicarbonate, antidote (fomepizole or ethanol), [15] folinic acid and dialysis are key components of treatment. Correctly diagnosed, early treatment can potentially save all victims, but availability of adequate facilities is variable and often scarce or totally lacking. The fact that methanol is often added to either illegal liquor[1, 8, 9] or even original bottles of spirit,[10] frequently creates the appearance of an epidemic. The number of victims within a short time-span often overwhelm resources. Further, lack of specific training for healthcare personnel and a delay in notification to the public, all play a role in the high fatality rates typically seen.[3]

Médecins Sans Frontières (MSF) operates in contexts where these poisonings are frequently seen. In 2013, a Memorandum of Understanding was signed between MSF and Oslo University Hospital (“The Methanol Poisoning Initiative”), aiming to quickly respond to suspected outbreaks, bringing experts of toxicology and intensive care as well as diagnostic tools, medications and simplified treatment protocols.

In Libya alcohol has been illegal to sell or consume since 1969, but there is a black market with smuggled and home-distilled alcohol. The outbreak described here was not unique; there were two additional smaller outbreaks during the previous six months (personal communication). Detailed descriptions in the international news are not found, but the Libya Herald reported “several deaths” last year in a report from March 2013.[16]

In Kenya the problem of illegal brewing and toxic alcohols is not new.[1, 17] Chang´aa is the most common locally brewed spirit. Sometimes methanol is used as a way of spiking the drink to give it an extra “kick”,[17] but most often used to dilute liquor for profit.[17]

Our aim in the present study was to describe findings from the first three large outbreaks of methanol poisoning where MSF participated, and evaluate the benefits of possible future collaboration between local authorities, a Non-Governmental Organisation (NGO) and international expertise. We are also presenting a new treatment protocol based upon local resource constraints that were used in the outbreaks (Attachment 1).

## Methods

This is a retrospective study of three major methanol poisoning outbreaks in Libya 2013, and Kenya, May and July 2014. De-identified data were collected from local MSF personnel, local health personnel, hospital files, and the media. The diagnosis was in most cases based on history and findings during the clinical examination. As regards to the history intake of alcohol and the geographical location, drinking together with other suspect victims etc. were important. During the clinical examination hyperventilation, mental status, visual disturbances, and whenever possible detection of metabolic acidosis were important for case definition. Retrospectively, analyses were done on some of the alcohol found in Kenya, where reportedly high methanol concentrations were found[18]. Formic acid was detected in some blood samples in Tripoli following establishment of the formate analysis during the MSF intervention.

All available data including time from initial case presentation to outbreak confirmation, response-time from local health authorities and MSF, diagnostics, medical treatment and outcomes were reviewed. Summary descriptive statistics were utilized.

### Ethics

The study was conducted retrospectively using previously obtained case reports from health facilities and media. No names or patient identifiers were collected. The study protocol was evaluated and approved by Ethics Review Board of MSF, Brussels, Belgium.

## Results

### Libya, March 2013

On March 7, several patients were admitted to different hospitals with a variety of clinical features (gastrointestinal symptoms, dizziness, dyspnoea, and visual disturbances) within Tripoli (estimated population 1.1 million). The following days, many more patients were hospitalized at Tripoli Medical Centre (TMC), Tripoli Central Hospital, Alkhadra Hospital, Istiqlal Hospital in Tajoura, Zahra Hospital, and Zawia Hospital. They had reportedly been drinking Bokha (often distilled from figs).[16, 19] Acute methanol poisoning was suspected based on clinical presentation only, as no methanol or formate analyses were available.

By March 11, hundreds of patients had been hospitalized.[20] On March 12, MSF in Libya notified the MSF Operational Centre in Brussels (MSF OCB) about the outbreak. The following day, the Department of Acute Medicine (Oslo University Hospital, OUH) was informed, and contact was made to MSF OCB through MSF Norway. An emergency medical team with experts in clinical toxicology and intensive care was established. MSF notified the Ministry of Health (MoH) and emergency visas were issued. MSF stocks of sodium bicarbonate and ethanol were increased concurrently. On March 15, the first doctor arrived with fomepizole, sodium bicarbonate, and a portable blood gas machine. Thirty-six hours later, two additional experts arrived with more medication and enzymes for establishing formate analysis locally.[19, 21]

Only three new patients were admitted following arrival of the MSF response team, all three were given fomepizole along with supportive treatment. There was initially a lack of antidote, but ethanol was made available to the hospitals after several days. Haemodialysis was available at TMC and Central Hospital. Information regarding treatment of the patients in Tripoli was limited, but regarding 88 patients (86 males, 2 females) admitted to TMC (which received a total of 415 cases), more comprehensive data were available: Their median age was 28 years (range 18–56), and the majority was admitted over a course of two days (31/88 on the 19^th^ and 46/88 on the 11^th^ of March). The most common presenting symptoms among these were GI symptoms (30/88; 34%), dizziness (21/88; 24%), visual disturbances (14/88; 16%), dyspnea (12/88; 13%) and “other” (11/88; 13%). They were generally acidotic, with a median pH of 7.21 (range 6.75–7.45), and their median bicarbonate was 8 mmol/L (range 2–37 mmol/L) (i.e. 8mEq (range 2–37 mEq). Most were hyperventilating with a median pCO_2_ of 2.7 kPa (1.1–7.3 kPa) or 21 mmHg (range 8–55 mmHg). Among these patients, 84/88 (96%) received bicarbonate in hospital, and 28/88 (32%) received hemodialysis). 65/88 (74%) survived without sequelae, 10/88 (11%) survived with sequelae, whereas 13/88 (15%) died.

**Fig 1:**
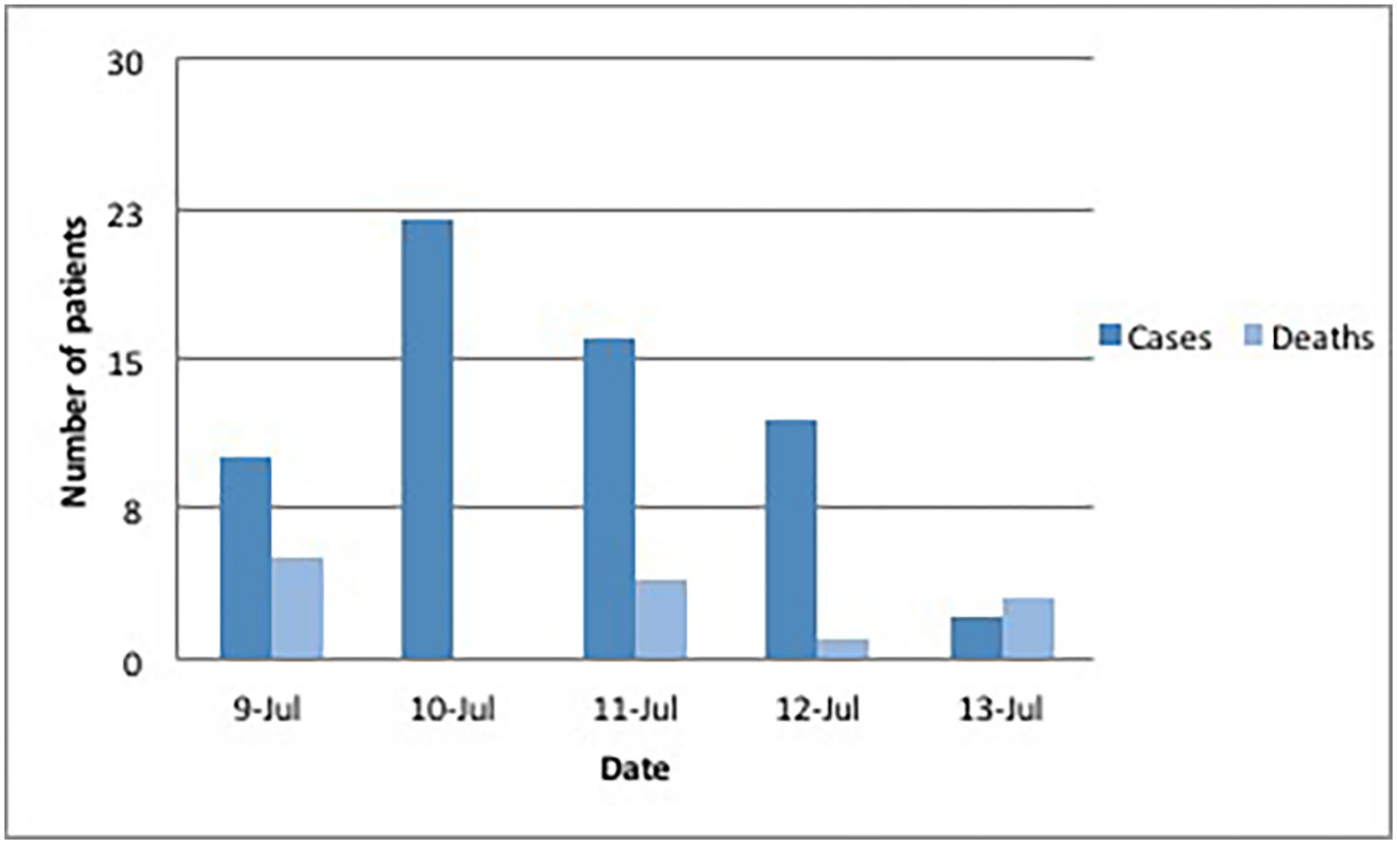
Frequency of methanol poisonings in Kapsabet, Kenya, July 2014. Source: MSF field report.

By March 26, a total of 1066 patients were reported, with 101 dead (case fatality rate = 10%, Fig 1 and Table 1) and 15 reported blind.[22] There were an unknown number of patients transferred to Tunisia, of whom 13 reportedly died during transport.[19]

**Table 1:** Number of methanol poisonings in Libya 2013 and Kenya 2014^a^.

Country		Poisonings	Deaths	Case Fatality Rate
Libya		1066	101	10%
Kenya	May	341	100	29%
	July	126	26	21%

^a^RE the source of data: All data were obtained through a triangulation from news, telephone inquiries to the various hospitals, and visits on site.

MSF and the MoH promptly arranged two large workshops at the university hospitals[19] including discussions on toxicology principles, diagnosis and treatment of methanol poisoning, based on locally adapted treatment protocols. A simple formate analysis was set up at TMC resulting in the only verified cases.[21]

### Kenya, May 2014

In May 2014, a large outbreak occurred in central Kenya.[23] The majority of patients were reported to have been drinking toxic alcohol on May 4. The first reports from multiple hospitals came on May 7. Media subsequently reported 60 dead and over 70 hospitalized in six counties.[23] Information collected by the local MSF team based on information from hospitals and media, concluded a total of 341 poisonings, of whom 100 died, case fatality rate = 29% (Table 1). The patients were mostly from small villages and slum areas. The toxic alcohol was found in both original sealed bottles and bootleg/homemade liquor. Diagnosis was based on history and clinical examination. None of the admitting hospitals had blood gas equipment, except for Kenyatta National Hospital (KNH) nor equipment to measure methanol, ethanol or formate. Ethanol was available for oral use in most hospitals (intravenous at KNH), however bicarbonate was rarely available. KNH was the only hospital with haemodialysis and an ICU, but with very limited capacity

#### The MSF intervention

MSF in Kenya was made aware of the outbreak on May 7. The following day, MSF OCB, MSF Norway and OUH decided to send a team of experts who left Oslo on May 9, arriving in Nairobi within 24 hours carrying additional medication and diagnostic equipment. Makueni and Embu hospital were chosen as interventional sites. A variety of equipment, medication (including ethanol) and diagnostics were brought to these hospitals. By the time of arrival, there were no new admissions and the focus turned towards patients already hospitalized and training workshops–arranged as a collaboration between MSF, KNH and the Kenyan MoH.

### Kenya, July 2014

On July 10–11, 2014 another flow of patients with symptoms consistent with methanol poisoning was reported from Kapsabet and Eldoret in western Kenya (Table 1).

In Kapsabet, toxic alcohol was consumed starting July 8. There were no specific diagnostics available, but after the third patient with similar symptoms, methanol poisoning was suspected. The media started to report about the incident later that day, and MSF Kenya established contact with the hospital (Fig 1).

Oral ethanol and intravenous sodium bicarbonate and folate were provided based on presenting symptoms and response to treatment. The hospital staff used a treatment protocol from the MoH, and had ethanol and bicarbonate available. Because of continued case presentations and falling supplies, the MSF team was dispatched from Nairobi on July 14 to Kapsabet (Fig 1), providing additional medications and oral alcohol. There were a total of 62 cases, of which 13 died (case fatality rate = 21%) (Table 1). Among these 62 cases, some additional data was obtained for 58 patients: There were 54/58 (93%) males (12 deaths (22%)) and 4/58 (7%) females (1 death (25%)). The median age was 30 years (range 21–62) among males, and 31 years (range 24–43) among females. Some patients reported drinking toxic alcohol from sealed 250ml bottles purchased at wine & spirits shops, whereas two patients reported drinking at a bar. The prompt response from local law enforcement officials to close all bars and wine & spirit shops in the area. This likely contributed to a rapid decline in cases. On July 15, an educational workshop was held by MSF for all available clinical staff at the hospital.

**Fig 2:**
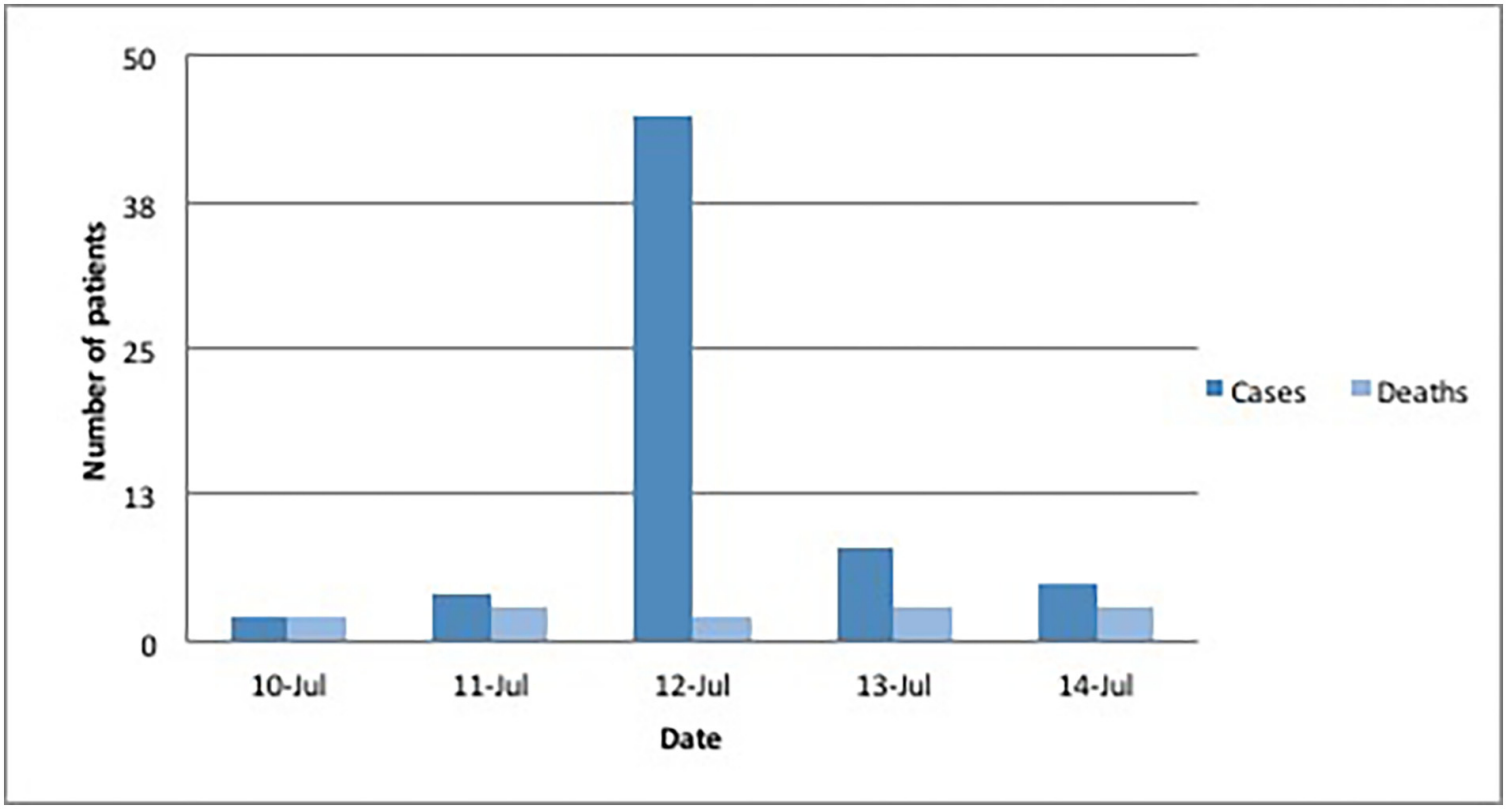
Frequency methanol poisonings in Eldoret, Kenya, July 2014. Source: MSF field report.

In Eldoret (population of 252 000) the first patients presented to the hospital on July 11 (Fig 2). All known cases were seen at Moi University Teaching and Referral Hospital (with eight ICU beds and eight dialysis machines). A total of 64 cases were received, of which 13 died (case fatality rate = 20%, Table 1). The MSF field team arrived on July 16, after which no new patients presented. The hospital treatment protocol was similar to the protocol of MSF/OUH. During the outbreak, the hospital ran out of pharmaceutical grade ethanol and utilized oral vodka.

According to police, most patients had been drinking in local bars, rather than alcohol bought at wine & spirits shops. Methanol poisoning diagnosis was made based upon on similar clinical symptoms after the first several patients. Public notification and closing of local bars by health officials and police were all likely reasons for the abrupt ending of the outbreak (Fig 2).

## Discussion

These three large methanol poisoning outbreaks highlight multiple public health challenges, including 1) symptom onset delay 2) symptom variability 3) lack of clinical experience 4) difficulties with diagnostic confirmation 5) limitations in treatment 6) overwhelming of health systems and 7) high case fatality rate. The limited awareness ultimately delays initiation of treatment and warnings to the public and other healthcare facilities. However, these challenges can be partially met by a joint effort between local health authorities, an NGO with the necessary infrastructure and emergency experience combined with international expertise providing detailed and locally adapted treatment protocols and training.

We describe the first three interventions by MSF and an expert toxicology team, where hundreds of patients were poisoned within a time frame of a few days. It clearly demonstrates how late recognition of the outbreaks delays the launching of the emergency teams, and as such, by the time of arrival the outbreaks were largely resolving. This emphasizes the three main reasons for the continued high morbidity and mortality in these poisonings: limited knowledge on the basic principles of methanol poisonings, the challenges in diagnosing such poisonings, and the difficulty in systematically treating a large number of patients with a high dependency level and significant resource constraints.

Whereas such outbreaks can last for weeks, months, or even years,[8, 10] the peak is typically in the earlier phases, highlighting the importance of an early identification and warning system which could initiate external help. Finally, the present study also documents the largest methanol outbreak ever reported, with more than 1000 patients being hospitalized within a few days in Libya.

In Libya, notification of possible poisonings reached MSF late, i.e. when the majority of patients had been admitted to a hospital or were already dead, but educational workshops were held while the outbreak was still on going.

This outbreak illustrates that even in countries where alcohol is banned, there remains significant risk for the harmful effects of methanol. Furthermore, there were reports of patients being reluctant to seek medical help and relatives not bringing affected family members for evaluation, because of perceived cultural, religious and legal consequences. This, along with the initial difficulties in obtaining ethanol for antidote purposes for an overwhelming number of patients, highlights some of the challenges facing treating physicians. A methanol outbreak within such a context likely has a significantly higher case fatality rate than what is reported.

Outbreaks in Kenya are well known from reports in the media over recent decades,[17] and there is a large illegal market for alcohol throughout the country. Additionally, it is common to “spike” drinks with methanol to increase potency,[17] paradoxically methanol itself has very limited intoxicating effects.[11] Many of the victims were poor and living in slum areas where healthcare is limited. These factors likely influenced the time to receive medical assistance.

It appears that delays from initial presentations to the general warning reaching the public and MSF, were likely related to a combination of lack of recognition and diagnostic capacity. This possibly contributed to a significant number of deaths, especially during the outbreak in May 2014. Thereafter, the response was swifter.

Thousands of bottles were however reportedly hidden away, making it possible for such epidemics to re-emerge. This is likely the cause of the second outbreak two months later. In Kapsabet, patients were more evenly spread out over five days, while in Eldoret most patients were admitted over 24-hours. This is most probably the direct result of a prompt response from law enforcement, halting all alcohol sales and the closing of bars locally. Lastly, during the outbreak in May, treatment varied and was not consistent from hospital to hospital, which indicates a general lack of standardized guidelines.

### What triggers the outbreaks?

According to WHO´s list of alcohol consumption per capita, countries such as Libya, Pakistan and Iran have very low consumption of alcohol.[24] However, when it comes to outbreaks of methanol poisonings these countries are often represented.[3] Thus, the illegal market may have a dominating role in places where alcohol consumption is forbidden and/or controversial, and controlling the content and spread may be even more challenging as was seen in Libya.

In countries where poverty is widespread, an illegal market is often triggered, which may include use of methanol. Also, in industrialized countries where the alcohol price is high, an illegal market and risk of methanol poisoning may occur.[8] Finally, regardless of the price of ethanol, bulk methanol can be bought less expensively, making outbreaks also appear in countries like Estonia,[9] Czech Republic,[10] Finland,[25] and Poland.[26]

### Main challenges

The outbreaks in this report demonstrate the typical delay from intake, to when awareness is triggered and treatment is initiated. A general lack of knowledge regarding methanol poisoning, leading to unawareness when patients are admitted with signs of a metabolic acidosis of unknown origin,[20] lack of diagnostic equipment,[2] and varying availability of treatment facilities[3, 9] all play a role. Methanol poisonings will most often be suspected when there are obvious outbreaks, and typically after a number of patients are already hospitalized or dead.[27] We have limited knowledge[25, 28] to what extent individual cases appear outside major outbreaks, but many cases are likely undetected due to lack of recognition and diagnostic tools.[20, 21]

### Suggestions for solution

Improving knowledge about toxic alcohols in general, and methanol poisoning in particular is important. Data collection and publishing reports from outbreaks in the medical literature would support this as well as establishing poison control centres with support from clinical toxicologists. Enlisting the assistance of organizations like MSF with collaborative projects, through open-access internet-based websites like WikiTox,[29] or the GetUp project by ACMT providing videoconferencing[30] can all contribute to improving the visibility and response to methanol poisoning.

Point of care diagnostics could dramatically reduce the delay of diagnosis, be crucial to initiating early treatment and mounting a swift public health response. This can be solved by a simple, disposable dipstick for accurate detection of formate, the toxic metabolite of methanol.[31] Fomepizole has now also been added to the WHO Essential Medicines List,[7] which should potentially improve availability and reduce cost.[15]

Simplified national treatment protocols, medical staff training and referral criteria to ICU-supported facilities would streamline and improve the quality of care in high-risk countries such as Kenya, Uganda, India, Pakistan, Cambodia, Vietnam and Indonesia. This increase in local competencies is crucial since mobilisation of international teams take time, and as demonstrated, external help may arrive after the peak of the outbreak, regardless, outbreaks may last for weeks, months or even years.[7, 9]

Active case finding^3^ is an under utilized method of limiting an outbreak where the objective is to find victims, prevent new cases by reaching the population and spreading information and warnings. This could include the use of mass media, social media, or via community leaders. If an outbreak takes place where drinking alcohol is not accepted, informing people that they will not be punished if seeking medical help, could be beneficial.

## Strengths and Limitations

The current study was retrospective and the exact case numbers were difficult to document. Caution must be made towards firm conclusions as regards to the total number of patients and mortality rates. However, we have used multiple different sources, including presence on site and the reports we have had access to are consistent with the over-all picture presented here. Due to lack of diagnostic tools, delay in admission, and some patients not being admitted, the numbers presented are more likely to be an underestimate rather than the opposite.

## Conclusion

Methanol outbreaks represent a significant challenge most often occurring in the developing world, frequently affecting poor and vulnerable populations. High case fatality rates for methanol poisonings in these contexts are likely secondary to large initial numbers of severely poisoned patients presenting rapidly, limited knowledge about methanol toxicology, diagnostic limitations and treatment constraints. Collaborations between local authorities, NGOs and international experts can improve outcomes within limited resource settings. Detailed reports from outbreaks worldwide are scarce and publication in medical literature of such information is strongly encouraged.

## Supporting Information

S1 File Simplified Treatment Protocol. This is based on experience and expert opinions only, solely meant for outbreak-situations and in situations where resources are limited.
(PDF)
