# Correction: The Methanol Poisoning Outbreaks in Libya 2013 and Kenya 2014

**DOI:** 10.1371/journal.pone.0157256

**Published:** 2016-06-03

**Authors:** Morten Rostrup, Jeffrey K. Edwards, Mohamed Abukalish, Masoud Ezzabi, David Some, Helga Ritter, Tom Menge, Ahmed Abdelrahman, Rebecca Rootwelt, Bart Janssens, Kyrre Lind, Raido Paasma, Knut Erik Hovda

The Acknowledgments section is incorrectly reported. The correct Acknowledgments section should read: We want to thank Dr. Rabie Mohammed Algdgad and Dr. Ahmed Bazza, Tripoli Medical Center for the contribution in collecting of data, and Prof. Michael Eddleston, Edinburgh, Scotland for his invaluable input and critical review of the manuscript. Study expenses were supported from the regular operation budgets of the MSF Kenya and Libyan missions as well as Oslo University Hospital, but had no role in the writing of the report as such.

## References

[1] Rostrup M, Edwards JK, Abukalish M, Ezzabi M, Some D, Ritter H, et al (2016) The Methanol Poisoning Outbreaks in Libya 2013 and Kenya 2014. PLoS ONE 11(3): e0152676 doi:10.1371/journal.pone.0152676 27030969 10.1371/journal.pone.0152676 PMC4816302

