# Supplementary material for: The Methanol Poisoning Outbreaks in Libya 2013 and Kenya 2014
Source: PLoS One. 2016 Mar 31;11(3):e0152676. doi: 10.1371/journal.pone.0152676 (PMC4816302; doi:10.1371/journal.pone.0152676)

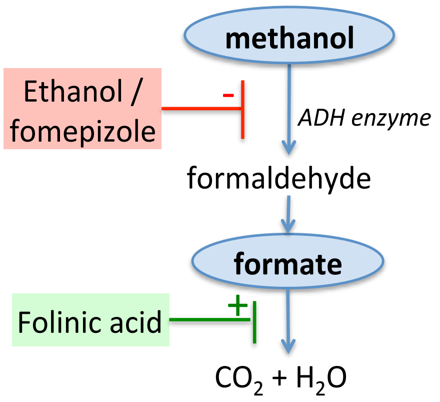

Methanol poisoning at a glance

Methanol is not toxic itself, but it is metabolized to the highly toxic formic acid/formate (see fig): The treatment is focused on blocking the enzyme (ADH) with either ethanol or fomepizole, buffer the metabolic acidosis with bicarbonate, and use dialysis to remove methanol, formate and correct the metabolic acidosis.
Folinic acid may also be given to enhance the endogenous metabolism of formate.
All of the above should be initiated as early as possible.

Diagnosis:

- **Symptoms**: hyperventilation/dyspnoea, visual disturbances (all kinds of), GI-symptoms, chest pain, "hangover".
- **Findings**: Arterial blood gas: Metabolic acidosis (unless concomitant ethanol intake), increased anion- and osmolal gap, increased serum-formate.

Treatment:

- Give **bicarbonate ($NaHCO_3$)** as soon as possible intravenously. Aim at full correction (0.3 x weight x base deficit (BD)) = mmol buffer (bicarbonate).
  (**or give 500 mmol if base deficit >20 over 1 hour, repeat if necessary**)
  If oral treatment: Tablets of 500 mg (= 6 mmol bicarbonate), 6-10 tablets every hour until acidosis/hyperventilation is corrected.

- Give **antidote** 1 or 2 without delay:
1. ***ethanol*** orally or intravenously – dosing: *See opposite side.*
2. ***fomepizole*** orally or intravenously – dosing: *See opposite side.*
   Duration of antidote: Give antidote until 12-24 hours after dialysis is finished, preferably ethanol in order to save fomepizole.

- **Dialysis (IHD-**intermittent, high-flow): Give for at least (6-) 8 hours if possible. **CVVHD**: 18hrs
- **Folinic acid** (or folic acid if folinic not available) 50mg iv or orally every 6 hours for 24-48 hrs
- **If ventilator support:** The patient must be hyperventilated as long as acidotic

Criterion for treatment (patients blood gases):

A. **Asymptomatic patients, normal blood gas**: Observe.
B. **pH>7.2, HCO_3>20:** Observe minimum 24 hours. Give bicarbonate if necessary (increasing acidosis)
C. **pH 7.0-7.2, HCO_3 10-20:** Give bicarbonate, ethanol (or fomepizole), consider HD
D. **pH<7.2, HCO_3<10:** Give bicarbonate, fomepizole (or ethanol), HD, folinic acid

- **RE antidote**:
  - *fomepizole* availability is limited; therefore, treat primarily only for the first 24 hours (two or max three doses per patient), then continue with ethanol after 24 hours
  - if dialysis is available immediately, give loading dose of fomepizole, then another dose after 4 hours of HD, then ethanol after the full course of HD (8 hours)
- **hemodialysis** (preferably intermittent, high flow (IHD). CVVHD if circulatory unstable, then >16 hrs)
  - consider in all patients with significant acidosis (pH <7.0-7.1, HCO_3<10) or visual disturbances. Always perform for at least 8 hours if possible to eliminate all methanol. If limited availability – consider alternating between patients with 2-3 hours interval

Prognostic aspects

Coma on admission, severe metabolic acidosis (pH<7.0) and lack of hyperventilation in spite of severe acidosis are the most prominent poor prognostic features on admission

Suggested dosing regimen for ethanol (be aware of individual differences and frequent under-dosing, i.e. check blood gases during treatment if available):

	5% ethanol	10% ethanol	20% ethanol	40% ethanol
**Loading dose**	15mL/kg	7.5mL/kg	4mL/kg	2mL/kg
**Infusion rate** (not regular drinker)	2mL/kg/hr	1mL/kg/hr	0.5mL/kg/hr	0.25mL/kg/hr
**Infusion rate** (regular drinker)	4mL/kg/hr	2mL/kg/hr	1mL/kg/hr	0.5mL/kg/hr
**Infusion rate during HD** (not regular drinker)	4mL/kg/hr	2mL/kg/hr	1mL/kg/hr	0.5mL/kg/hr
**Infusion rate during HD** (regular drinker)	6mL/kg/hr	3mL/kg/hr	1.5mL/kg/hr	0.8mL/kg/hr

If Serum-ethanol analyses are available: aim at serum-ethanol 100-150mg/dL

Suggested dosing of fomepizole:

- Normal dosing:
  - Loading dose 15mg/kg, then
  - 10mg/kg every 12 hour (every 4 hours during HD)
- Dosing during outbreak with limited capacity or availability of fomepizole:
  - Loading dose 10mg/kg, then
  - 10mg/kg every 12 hour (every 4 during HD) for the first 24 hours, then use ethanol to save fomepizole
  - Suggested use of ethanol also after discontinuation of HD (6-8 hours of HD) to save fomepizole

Formate analysis - use in clinical setting:

If no formate is produced from methanol poisoning, no symptoms occur, i.e. ANY patients with a metabolic acidosis because of a methanol poisoning must have traceable formate:

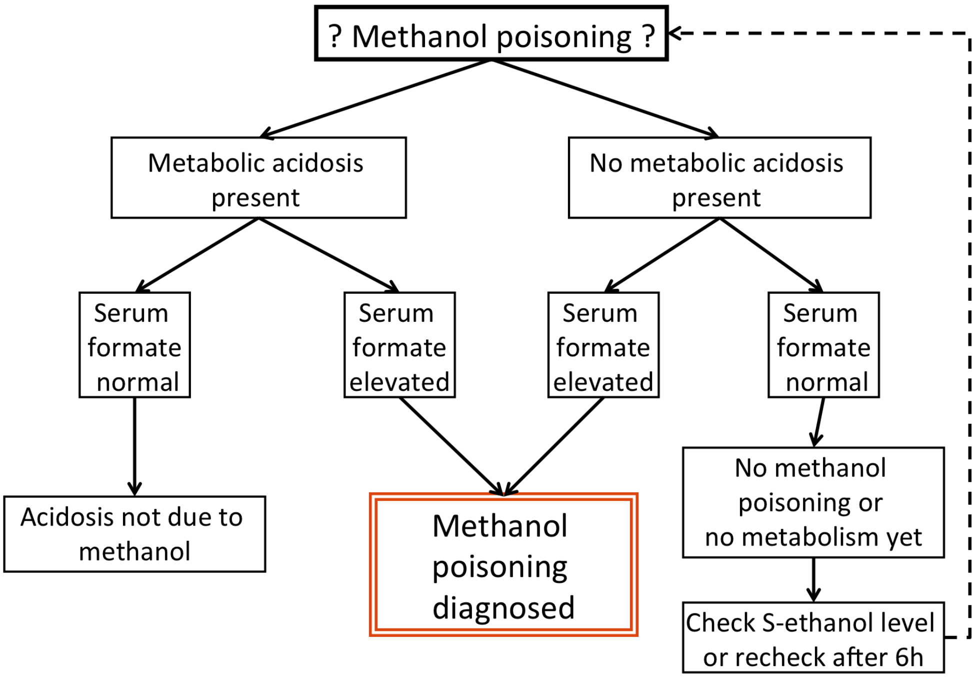

Supplement: S1 File — This is based on experience and expert opinions only, solely meant for outbreak-situations and in situations where resources are limited. (PDF) [file pone.0152676.s001.pdf]
